# A two-layer mono-objective algorithm based on guided optimization to reduce the computational cost in virtual screening

**DOI:** 10.1038/s41598-022-16913-w

**Published:** 2022-07-27

**Authors:** Miriam R. Ferrández, Savíns Puertas-Martín, Juana L. Redondo, Horacio Pérez-Sánchez, Pilar M. Ortigosa

**Affiliations:** 1 Supercomputing-Algorithms Research Group (SAL), Agrifood Campus of International Excellence, University of Almería, Carretera Sacramento s/n, La Cañada de San Urbano, 04120 Almería, Spain; 2 Structural Bioinformatics and High Performance Computing Research Group (BIO-HPC), HiTech Innovation Hub, Universidad Católica San Antonio De Murcia (UCAM), Campus de los Jerónimos, 30107 Murcia, Spain

**Keywords:** Drug discovery, Virtual drug screening, Mathematics and computing

## Abstract

Virtual screening methods focus on searching molecules with similar properties to a given compound. Molecule databases are made up of large numbers of compounds and are constantly increasing. Therefore, fast and efficient methodologies and tools have to be designed to explore them quickly. In this context, ligand-based virtual screening methods are a well-known and helpful tool. These methods focus on searching for the most similar molecules in a database to a reference one. In this work, we propose a new tool called 2L-GO-Pharm, which requires less computational effort than OptiPharm, an efficient and robust piece of software recently proposed in the literature. The new-implemented tool maintains or improves the quality of the solutions found by OptiPharm, and achieves it by considerably reducing the number of evaluations needed. Some of the strengths that help 2L-GO-Pharm enhance searchability are the reduction of the search space dimension and the introduction of some circular limits for the angular variables. Furthermore, to ensure a trade-off between exploration and exploitation of the search space, it implements a two-layer strategy and a guided search procedure combined with a convergence test on the rotation axis. The performance of 2L-GO-Pharm has been tested by considering two different descriptors, i.e. shape similarity and electrostatic potential. The results show that it saves up to 87.5 million evaluations per query molecule.

## Introduction

Nowadays, the COVID-19 pandemic is highlighting the urgent need to speed up the discovery procedure for new drugs^1^. One of the main problems in this process is the number of molecules to be analyzed. Their information is stored in databases that contain information such as the number of atoms and bonds, their 3D position, electrostatic charge, among others, and can contain up to millions of molecules. In this context, some computer-aided techniques are becoming more popular, such as Virtual Screening (VS)^2,3^. The main idea of VS is to process an existing database of approved compounds to search for molecules of interest. Once this in-silico pre-filter is carried out, only those relevant molecules are studied experimentally in the laboratory. Accordingly, to guarantee the success of VS, its mechanisms should be accurate enough to identify promising compounds within huge databases and, at the same time, fast enough to process all the compounds in affordable computing times.

First of all, among VS methods, we must distinguish between Structure-Based VS (SBVS) or Ligand-Based VS (LBVS)^4^. In SBVS, the protein target has a known structure. Some SBVS methods are molecular docking^5^ and molecular dynamics^6^. When the protein target structure is not available, LBVS methods are employed since they focus on certain desirable properties of known compounds^7^. In our work, we focus on the LBVS family of methods called similarity methods. In these similarity methods, given a molecule named as query, the objective is to find the most similar molecule among the set of molecules (referred to as targets) from a given database. The similarity between the query and a target can be measured by paying attention to different descriptors such as, for instance, their shapes or their electrostatic potential.

According to the state-of-the-art, concerning shape similarity, the reference algorithms are the Weighted Gaussian Algorithm (WEGA)^8–10^ and Rapid Overlay of Chemical Structures (ROCS)^11,12^. In particular, the latter is the most referenced and widely used algorithm in the VS context^13^. However, WEGA proved in^8,9^ to be better than ROCS in terms of accuracy of the results. Recently, a new piece of software, called OptiPharm, proving to be competitive against WEGA, has been proposed in the literature^14,15^. As far as we know, and as was demonstrated in^14^, the version of OptiPharm is the only software that performs simultaneously both shape and electrostatic similarity. In addition to this, among other advantages, OptiPharm shows the same predictive accuracy than WEGA but at a much lower computational cost^14^. Moreover, regarding electrostatic similarity, OptiPharm demonstrated to be an excellent alternative to maximize the electrostatic similarity between molecules versus the traditional methodology followed. For all those reasons, we consider OptiPharm as the reference algorithm to compare and try to beat by saving in the number of evaluations and, thus, reducing the computational cost.

To give a general idea, OptiPharm^14,15^ is a global evolutionary optimization algorithm designed to compute the maximum similarity between any query-target pair of compounds. More precisely, it is a population-based optimizer, in the sense that it starts with a set of candidate solutions, known as individuals, and applies a series of mechanisms to modify them such that they evolve towards the optimum solution. In OptiPharm, any solution or individual is composed of ten variables, namely the rotation angle, two three-dimensional coordinates determining the rotation axis, and three translation values giving the displacement in each axis. Moreover, as the main feature of the algorithm, each individual in the population has a radius value that is used to focus the search within the neighborhood bounded by this radius.

In this work, we propose a new algorithm as an alternative to OptiPharm. It is called 2L-GO-Pharm since it is based on Guided Optimization and is organized into two layers. Similarly to OptiPharm, it works with a population of individuals that evolve iteratively. However, as a novelty, their changes are guided by a leader solution, which is the one obtaining the best score in each iteration. Moreover, in 2L-GO-Pharm, individuals are composed of only six variables using a semi-sphere parametrization that simplifies the definition of the rotation axis and provides it with uniqueness. As a consequence, the searchability is enhanced due to the reduction of the search space dimension and the fact that we avoid the repetition of proportional vectors determining the same axis. Apart from that, the two-layer strategy of 2L-GO-Pharm guarantees a balance between exploration and exploitation of the search space. The first stage of the algorithm attempts to detect new solutions that have the potential to become local or global optima. We can think of it as an exploration level aimed at achieving specific solutions that we will use in the second layer as individuals for the initial population. The second layer is an exploitation stage, aimed to move the solutions toward the peaks, looking for accuracy in the results. This new design allows us to reduce function evaluations that translate to saving time, but without lack of quality on the solutions proposed. In addition to that strategy, it also incorporates some problem knowledge, such as a mechanism to keep the angular variables between 0 and $2\pi$ in a continuous circular way, and a convergence test to maintain the exploitation of a particular rotation axis when convergence is not reached, or if not, to explore other axes.

As the results will show, this new way of approaching the problem with 2L-GO-Pharm allows predictions similar and/or better to those proposed by OptiPharm to be found but performing a smaller number of evaluations. More precisely, for two different databases that are composed of 1750 molecules and 28,374 molecules, the total savings in evaluations are 87.5 million and 6418.7 million for each query, respectively. This contribution is not only relevant in the current context, where reducing the computation time is a desirable requirement, but it can become even more important in VS problems where the molecules are flexible, since the databases are even larger (see^16–19^).

Additionally, from an optimization point of view, a previous analysis of the features of the function (or functions) to optimize is highly recommended. This paper also includes a brief resume of the analysis we have carried out. It highlights how difficult it can be to find the global optimum of the electrostatic similarity, as it is a non-smooth function with a significant number of peaks. Then, the algorithms can be easily trapped in local optima. However, 2L-GO-Pharm has been specifically designed to avoid getting stuck in local optima by incorporating its two-layer strategy. Consequently, the results obtained in electrostatic similarity show that the 2L-GO-Pharm improvements in terms of the quality of the solution are quite noticeable and significant. Therefore, 2L-GO-Pharm is the best option when the complexity of the function or descriptor is challenging or not known.

The rest of the article is organized as follows. In “Materials and methods” section, we introduce the shape and electrostatic similarity notions and computations, and the decision variables considered for the mono-objective problems needed to maximize those similarities. We also detail the optimization algorithm 2L-GO-Pharm that we have designed to solve those problems. Then, in “Computational experiments framework” section, we explain the computational experiments carried out to prove the performance of the proposed optimization algorithm and to compare it with the state-of-the-art algorithm, OptiPharm. We also include all the information required to reproduce those experiments, such as settings of the algorithms, compounds databases, and metrics. After that, in “Results and discussion” section, we present and discuss the obtained results. Finally, in “Conclusions” section, we highlight the main conclusions and provide an outlook regarding future research directions.

## Materials and methods

In this section, we introduce the basic foundations of our work: the mono-objective optimization approach (see “Mono-objective optimization problems” section) and the novel optimization algorithm developed to deal with it (see “Optimization algorithm 2L-GO-Pharm” section).

### Mono-objective optimization problems

VS is aimed at finding the target compound most similar to a given query molecule. The similarity of a query-target pair can be quantified by paying attention to different descriptors^20^. In this work, we focus on two particular descriptors: shape similarity and electrostatic similarity.

More precisely, in this work, we tackle each descriptor separately, such that we deal with the maximization of the shape similarity and electrostatic similarity as independent problems. Accordingly, each of them is a mono-objective problem whose objective function is the similarity score.

In “Shape similarity” and “Electrostatic similarity” sections, we introduce the concepts of shape similarity and electrostatic similarity, respectively, and we present the mathematical formulas used to quantify them. In “Decision variables” section, we define the variables used to describe the position of the target molecule in the 3-dimensional space. Those variables act as decision variables in the mono-objective problems. Finally, in “Smoothness of the objective functions” section, we perform a brief graphical analysis of both shape and electrostatic similarity functions.

#### Shape similarity

The shape similarity between two molecules is defined as the overlapping volume of their atoms. To compute this overlapping volume, several approaches are mentioned in the literature. The most popular are ROCS^11–13^ and WEGA^8–10^. In this case, we use the formulation proposed in WEGA^8,9^, since it incorporates a weight factor for each atom in first-order terms of the molecules’ density function, enhancing the accuracy.

Given two molecules denoted by *A* and *B*, we consider their atoms denoting them with the indexes *i* for the atoms of molecule *A* and *j* for those of *B*, i.e. $i\in A$ and $j \in B$. As such, their overlapping volume $V_{AB}^{g}$ is obtained as follows:

1 $$\begin{aligned} V_{AB}^{g} = \sum _{i\in A, j \in B} w_i w_j v_{ij}^g, \end{aligned}$$

where $w_i$ and $w_j$ are weights corresponding to the atoms *i* and *j*, respectively. Those weights are computed using the following formula:

2 $$\begin{aligned} w_i = \frac{v_i}{v_i + k \sum _{j\ne i} v_{ij}^g}, \end{aligned}$$

where $k=0.8665$ is a universal constant, and $v_i$ is the volume of the atom *i*, which is calculated using the volume of the sphere as in^8^, $v_i=\frac{4\pi \sigma _i^3}{3}$, $\sigma _i$ being the radius of the atom.

In Eq. (1), the superindex *g* indicates that the Gaussian representations are used to compute the overlapping between atoms as follows:

3 $$\begin{aligned} v_{{ij}}^{g} & = \iiint {g_{i} (r)g_{j} (r)d{\mathbf{r}}} \\ & = \iiint {pe^{{ - \left( {\frac{{3p\pi ^{{1/2}} }}{{4\sigma _{i}^{3} }}} \right)^{{2/3}} }} (r - r_{i} )^{2} pe^{{ - \left( {\frac{{3p\pi ^{{1/2}} }}{{4\sigma _{j}^{3} }}} \right)^{{2/3}} }} (r - r_{j} )^{2} {\mathbf{dr}},} \\ \end{aligned}$$

where *p* is a parameter controlling the softness of the Gaussian spheres, $r_i$ is the position of atom *i*, and the radius of the atom $\sigma _i$ represents the well-known van der Waals radius. Those parameters are set to empirical values as in^8^.

Notice that Function 1 obtains a different range of values depending on the size of the molecules. Consequently, these values must be normalized to compare results. Some works can be found in the literature with varying metrics on this subject, such as Tversky^21^ or Tanimoto^22^ coefficients. For this work, it has been decided to use the latter to normalize the values to compare different molecules. The Tanimoto coefficient, widely used in virtual screening, is based on calculating the similarity of two sets. Whether the intersection of both sets is one of the sets (because they are equal), the similarity value is 1. This value decreases as there are differences between both sets until it reaches 0, where there is no intersection. Adapted to our problem and as used by other software^8,13^, this coefficient is defined as follows:

4 $$\begin{aligned} Tc_{S} = \frac{V_{AB}^g}{V_{AA}^g+V_{BB}^g-V_{AB}^g}, \end{aligned}$$

such that $T_c \in [0,1]$, where 0 means no overlapping and 1 means that the molecules have the same shape densities.

#### Electrostatic similarity

Electrostatic potential $\phi (r)$ is related to the molecular charge distribution $\rho _{\mathrm{mol}}(r)$ through the following equation:

5 $$\begin{aligned} \nabla \{ \varepsilon (r) \nabla \phi (r)\}=-\rho _{\mathrm{mol}}(r), \end{aligned}$$

where $\varepsilon (r)$ is the dielectric constant. This equation is known as the Poisson equation, and it is solved numerically since it is a second-order Partial Differential Equation (PDE).

Accordingly, given two compounds *A* and *B*, their electrostatic similarity is measured by computing:

6 $$\begin{aligned} E_{AB}=\int \int \int \phi ^A(r)\phi ^B(r) \Theta ^A(r) \Theta ^B(r) \mathbf {dr} \approx h^3 \sum _{i,j,k} \phi _{ijk}^A\phi _{ijk}^B \Theta _{ijk}^A \Theta _{ijk}^B, \end{aligned}$$

where $\Theta$ is a masking function to guarantee potentials within the compound are not considered part of the comparison. Notice that the volume integral involved is approximated using a spatial mesh with grid-spacing parameter *h*.

Analogously to the shape similarity, we use the Tanimoto similarity to avoid dependence on the number of atoms of the compared molecules:

7 $$\begin{aligned} Tc_{E} = \frac{E_{AB}}{E_{AA}+E_{BB}-E_{AB}}, \end{aligned}$$

such that $Tc_{E} \in [-0.33, 1]$, where $-0.33$ means molecules with the same charge value but opposite loads, 0 indicates that there is no overlapping, and 1 corresponds to compounds with the same charge.

#### Decision variables

The above-defined descriptors of the similarity between two molecules depend on their relative positions. Thus, for each query-target pair, a mono-objective problem is defined to find the configuration giving the maximum similarity. In this search, we assume that the query molecule is fixed, so we use the following six variables to describe the position of the target:

$$\begin{aligned} \alpha , \theta , \varphi , \Delta x, \Delta y, \Delta z, \end{aligned}$$

where $\alpha \in [0, 2\pi ]$ is the rotation angle, $(\theta , \varphi ) \in [0, 2\pi ]\times [0, \pi /2]$ are the spherical coordinates of a unitary vector defining the rotation axis, and $\Delta x, \Delta y, \Delta z$ are the translations in the *X*, *Y* and *Z* directions, respectively.

**Figure 1:**
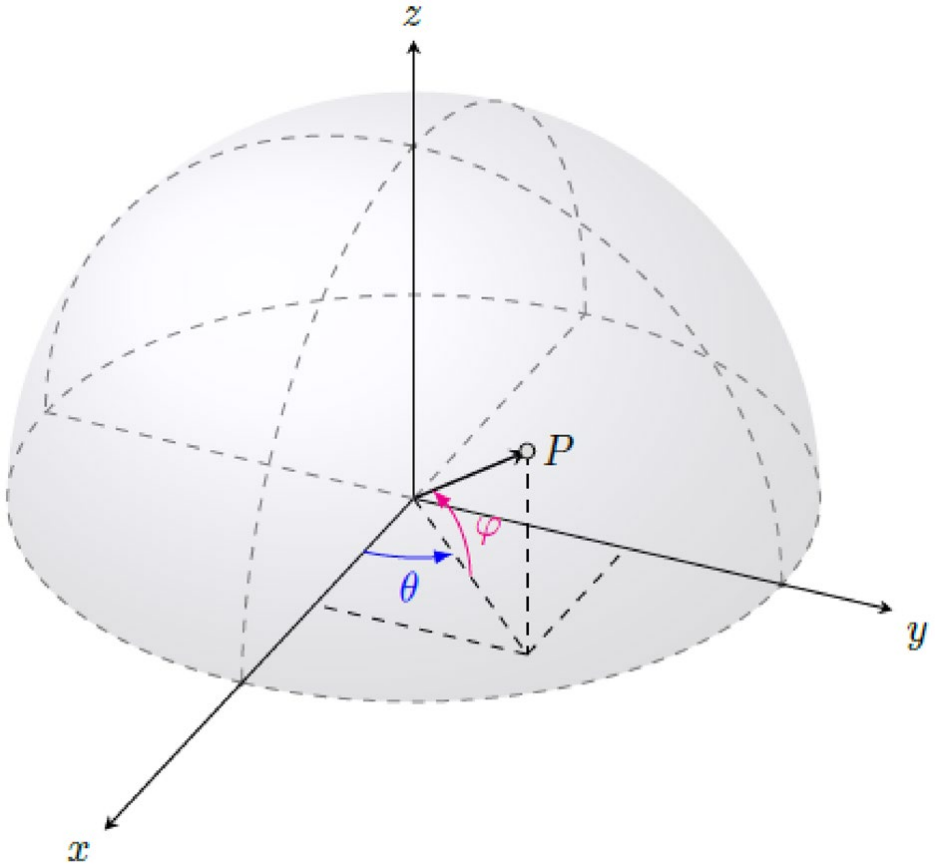
Spherical coordinates $(\theta , \varphi ) \in [0, 2\pi ]\times [0, \pi /2]$ of a unitary vector $\overrightarrow{OP}$ to define the rotation axis.

Note that to avoid repeating proportional vectors determining the same axis, we define the rotation axis by a unitary vector using the semisphere parametrization:

$$\begin{aligned} x=\cos (\theta )\sin (\varphi ), \quad y=\sin (\theta )\sin (\varphi ), \quad z=\cos (\varphi ), \end{aligned}$$

where $\theta \in [0, 2\pi ]$ and $\varphi \in [0, \pi /2]$. In Fig. 1, those decision variables $\theta$ and $\varphi$ and their search domain, which is the semisphere, are represented.

**Figure 2:**
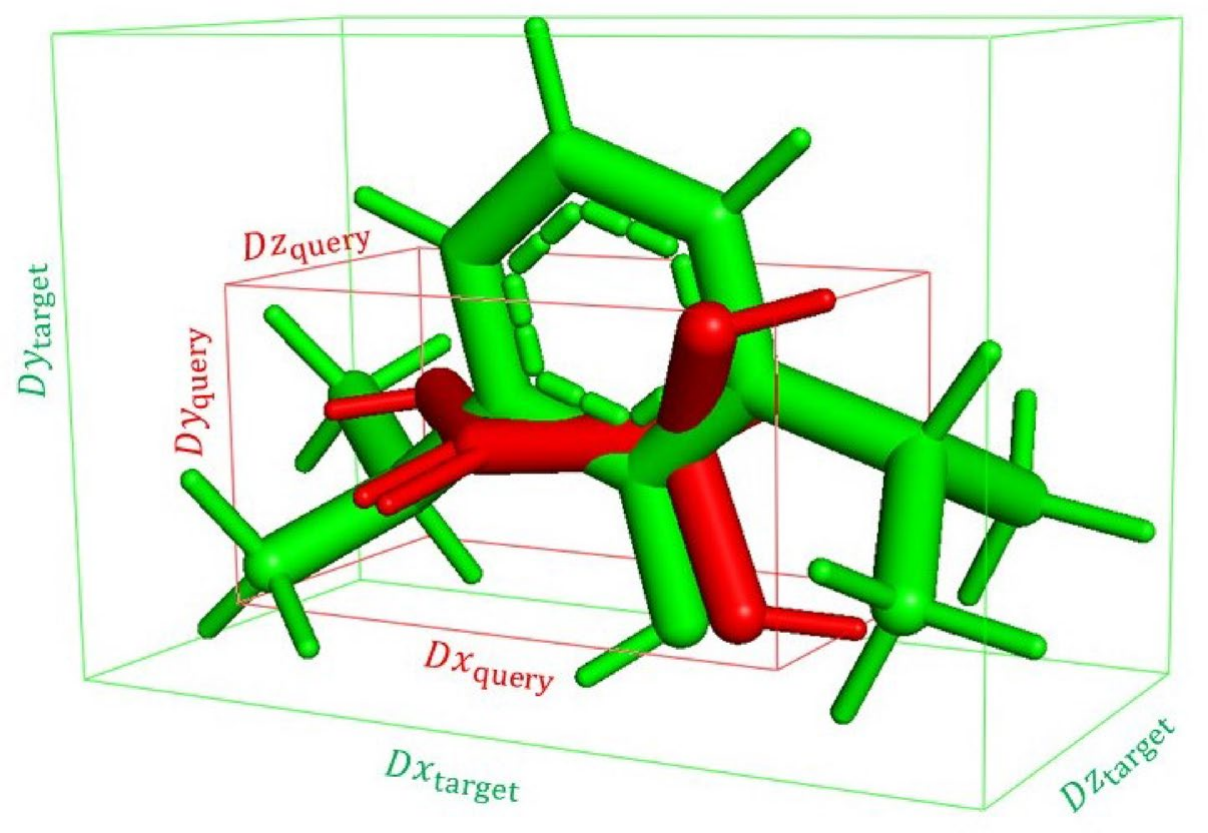
Boxes containing the query (in red) and the target (in green) compounds.

The displacements $\Delta x, \Delta y, \Delta z$ are bounded by $[-max_X, max_X],$ $[-max_Y, max_Y],$ $[-max_Z, max_Z]$, respectively, $max_X, max_Y, max_Z$ being the maximum difference on each axis *X*, *Y*, *Z*, between the boxes that contain the molecules^14^. In Fig. 2, we show a graphical example for the query DB00529 (coloured in red) and the target DB00818 (coloured in green), both from the U.S. Food and Drug Administration (FDA) database^23^.

Thus, the search domain $\Omega \subset {\mathbb {R}}^6$ for the optimization problem, is

$$\begin{aligned} \Omega = [0, 2\pi ] \times [0, 2\pi ] \times [0, \pi /2] \times [-max_X, max_X] \times [-max_Y, max_Y] \times [-max_Z, max_Z]. \end{aligned}$$

#### Smoothness of the objective functions

When dealing with an optimization problem, it is important to undertake a previous analysis of the features of the objective function that we want to optimize. For instance, studying the smoothness of the function may help us to decide which approaches are suitable for its optimization. We have therefore carried out a graphical analysis of both shape and electrostatic similarity functions. Since the behaviour is similar for all the query-target pairs, we illustrate here a particular case where the query compound is DB00381 and the target DB01023, both from the FDA database^23^.

**Figure 3:**
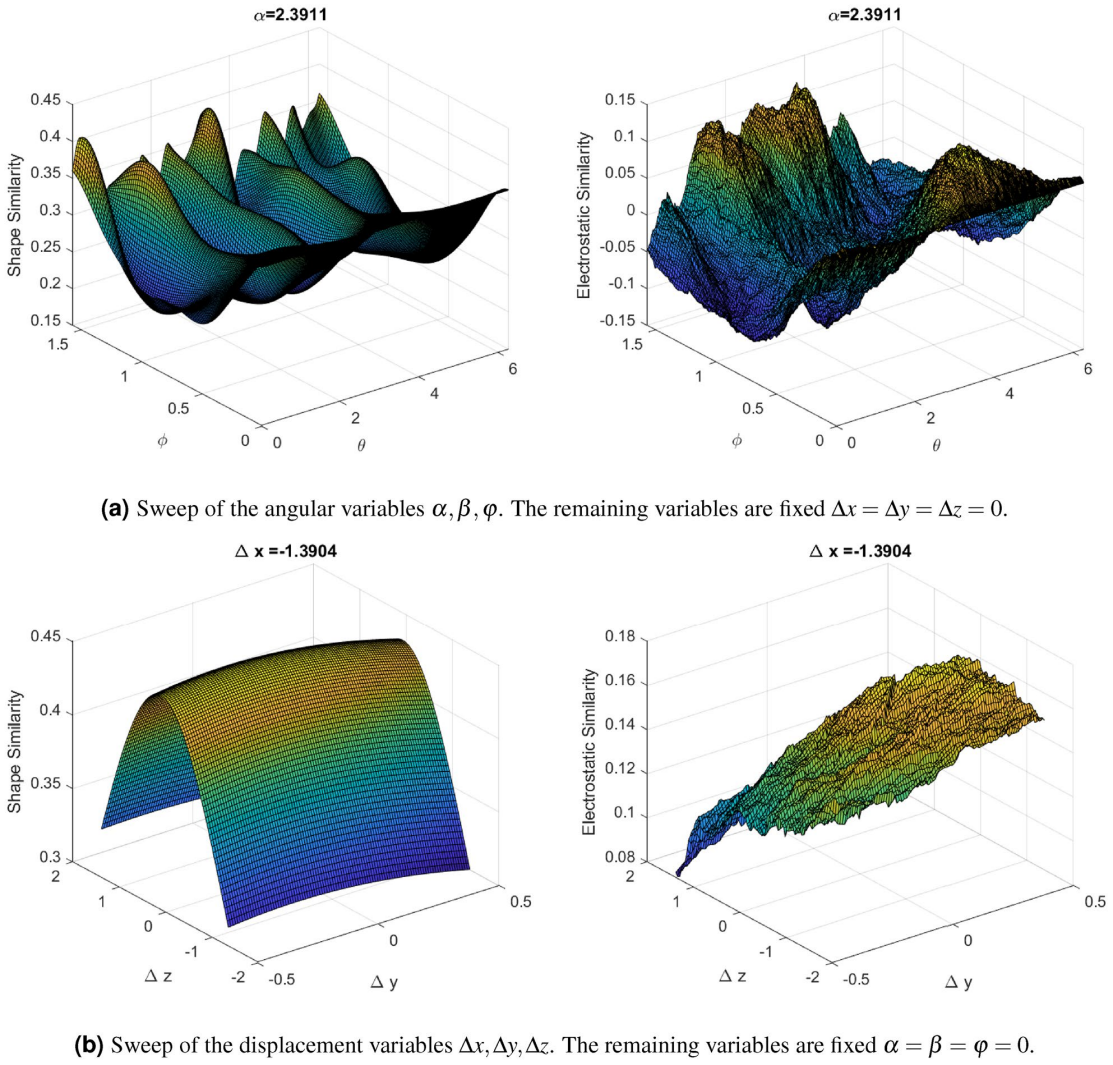
Representation of both objective functions, shape similarity (left) and electrostatic similarity (right), for certain sweeps of the decision variables. In this case, the considered query and target compounds are DB00381 and DB01023, respectively.

To obtain a 3D-plot representation of each function, as we consider six decision variables (see “Decision variables” section), we evaluate each function by simultaneously varying three variables in a sweep where the other three fixed are kept fixed. In Fig. 3a, we show a step of the sweep in which we change the values of the angular variables $\alpha , \beta , \varphi$ within their ranges while keeping the displacement variables at zero. Analogously, in Fig. 3b, we show a step of the sweep where we vary the displacement variables values $\Delta x, \Delta y, \Delta z$ within their ranges while keeping the angular variables at zero.

As can be observed in Fig. 3, the shape similarity function (on the left-hand side) seems to be a smooth function that may present several local optima but on a smooth surface, in the sense that it is a differentiable surface. However, the electrostatic similarity function (on the right-hand side) is non-smooth, i.e., non-differentiable, since it exhibits peaks on its surface. As a consequence, from an optimization point of view, the electrostatic similarity global optimum is more difficult to achieve because the algorithms can be easily trapped in local optima.

### Optimization algorithm 2L-GO-Pharm

Based on the knowledge regarding the problem, we have designed an optimization algorithm called 2L-GO-Pharm composed of two layers. In each layer of 2L-GO-Pharm, we execute a mono-objective evolutionary algorithm named GO-Pharm, also proposed as a novelty in this work, to optimize the similarity between the molecules starting with different initial poses. In “Mono-objective algorithm GO-Pharm” section, we present the mono-objective algorithm GO-Pharm and explain in detail its parameters and phases. Then, in “Two-layer strategy” section, we focus on the two-layer strategy of 2L-GO-Pharm.

#### Mono-objective algorithm GO-Pharm

The mono-objective algorithm GO-Pharm, which we propose here, is a Guided Optimization algorithm designed to deal with the similarity mono-objective problems that appear in the pharma industry. More precisely, it is a population-based evolutionary optimizer where a leader solution guides a population of candidate solutions called individuals. Although this idea is inspired by the Teaching-Learning-Based Optimization (TLBO) algorithm^24^, GO-Pharm incorporates several new additional mechanisms and some problem knowledge, as detailed below.

**Figure 4:**
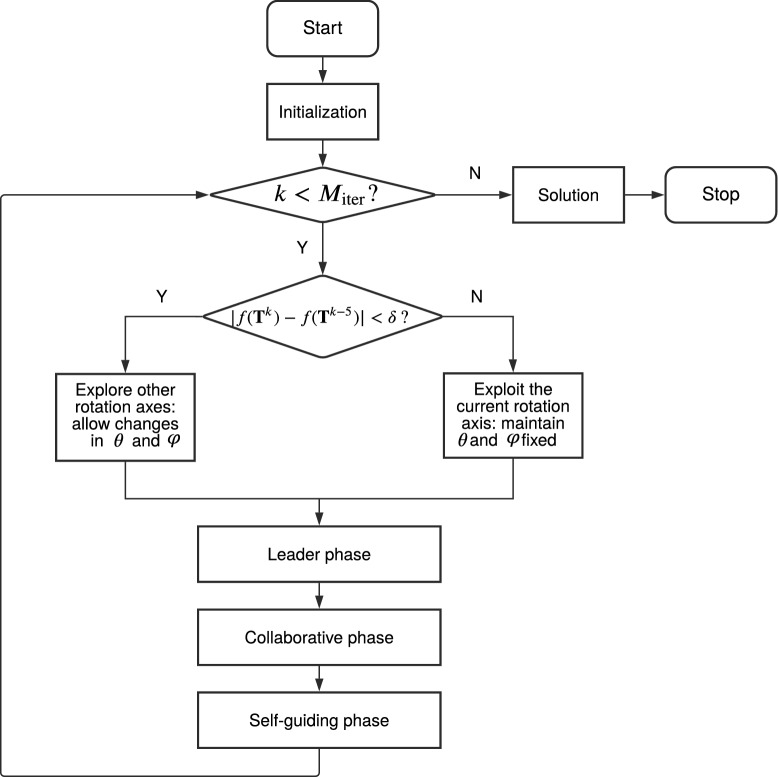
Flux diagram of GO-Pharm.

Now, we can describe in depth the GO-Pharm structure, highlighting its main features and novelties. In Fig. 4, we show the flux diagram of the algorithm. For the sake of completeness, in Algorithm 1, we include detailed pseudocode of GO-Pharm.

In terms of input parameters, it receives the size of the population denoted by *N*, the maximum number of iterations designated by $M_{\mathrm{iter}}$, a tolerance value denoted by $\delta$, and a polynomial mutation parameter designated by $n_m$. The first two parameters are specific to the original TLBO algorithm. However, for the current problem, we incorporate the two latter parameters in GO-Pharm. The tolerance value $\delta >0$ is employed in a novel mechanism that we introduce in GO-Pharm to improve searchability and to maintain a balance between exploration and exploitation. The polynomial mutation parameter $n_m>0$ is used in two polynomial mutation operators within the leader and self-guiding phases (see Fig. 4) to explore the search space in the neighborhood of a promising individual within the population^25^.

As previously mentioned, GO-Pharm works with a population of candidate points or individuals of the search space that we define as $P^k=\{{\mathbf {X}}^k_i\}_{i=1,\dots ,N}$, where the superindex *k* denotes the iteration and the subindex *i* refers to the *i*-th individual in the population. The decision variables of a particular individual ${\mathbf {X}}^k_i$ are denoted by $X^k_{ij}$, where $j \in \{1,2,\dots ,D\}$ and *D* is the number of decision variables, i.e., ${\mathbf {X}}^k_i=(X^k_{i1}, X^k_{i2}, \dots , X^k_{iD})$. The lower and upper boundaries of each variable are denoted by $L_j$ and $U_j$, respectively, such that $L_j\le X^k_{ij}\le U_j$, $j \in \{1,2,\dots ,D\}$.

As shown in Fig. 4, GO-Pharm begins with an initialization stage which sets and evaluates an initial population $P^0=\{{\mathbf {X}}^0_i\}_{i=1,\dots ,N}$ of *N* individuals. As in TLBO, they can be randomly generated. However, in GO-Pharm, as a novelty, we also implement the option of including certain initial points that are good candidate solutions because they have been obtained as optimum solutions in previous procedures or because of the problem knowledge, such as some rotations in the cartesian axes (see “Two-layer strategy” section).

As can be seen in Fig. 4, after initialization, an evolutive procedure starts where the individuals of the population are modified with the aim of improving them in each iteration $k>0$, until reaching the maximum number of iterations $M_{\mathrm{{iter}}}$. When all the $M_{\mathrm{iter}}$ iterations are completed, the final solution of GO-Pharm is then obtained as the individual in the population with the best score for the objective function.

As long as the maximum number of iterations is not reached, at each iteration $k>0$, $k=1,\dots ,M_{\mathrm{iter}}$, a convergence test is carried out by computing the difference in absolute value between the optimal value of the objective function obtained five iterations before and the one achieved in the current iteration (see Fig. 4). If it is less than the considered tolerance $\delta >0$, then convergence is accepted and other rotation axes are explored by allowing modifications in the axis variables $\theta$ and $\varphi$ in the subsequent phases. Otherwise, if convergence is not reached or this test cannot be performed because $k<5$, then we continue the exploitation of the current rotation axis by maintaining the current values of $\theta$ and $\varphi$ in the phases that follow. This convergence test is a novel feature of GO-Pharm and it is inspired by the idea that in the search for the position of maximum similarity, once the axis is fixed, the algorithm can focus on looking for some appropriate rotation angle and translation vector. To do that, the axis variables $\theta$ and $\varphi$ are fixed until certain convergence is reached by modifying the remaining variables.

After the convergence test, we consider the population from the previous iteration $P^k=P^{k-1}$ and try to improve it through three stages (see Fig. 4): (1) the leader phase, where the individual with the best score on the objective function guides the other individuals from the population in the search space; (2) the collaborative phase, in which the individuals share knowledge between them; (3) the self-guiding phase, where each individual can mutate by itself. The leader phase was originally proposed in TLBO^24^. Nevertheless, in GO-Pharm, we incorporate a polynomial mutation operator following the ideas in^25^ to take advantage of the origin-bias that arises in TLBO. Furthermore, to enhance the guided optimization strategy inspired by TLBO, included in GO-Pharm is the self-guiding phase as a novel extra phase where another polynomial mutation operator is used to increase the diversity of the population and avoid a premature convergence towards a local optimum.

All those three phases represented in Fig. 4 follow a similar structure: firstly, new candidate individuals for the population are obtained from the previous individuals; secondly, a projection operator denoted by $Proj_{\Omega }({\mathbf {X}}_i)$ is applied to keep the decision variables inside the search domain $\Omega = [L_1,U_1] \times \dots \times [L_D,U_D]$; then, those candidate individuals are evaluated using the objective function; and, finally, they are accepted to replace the previous individuals in the population if they have a better score on the objective function.

Concerning the projection operator, we must highlight another important innovative feature of GO-Pharm related to the problem knowledge: the circular limits. The main idea of the circular limits is to give search continuity to those angular variables whose search domain is $[0, 2\pi ]$ (see^26^). In this regard, if any of these variables takes a value above 2$\pi$ or below 0, then it is translated to an angle in the interval [0, 2$\pi$] by computing its 2$\pi$ module. In our problem, it is the case of the angles $\alpha$ and $\theta$ related to the rotation of the molecule (see “Decision variables” section). Therefore, the considered projection function is $Proj_{\Omega }({\mathbf {X}}_i)=(Proj_{[L_1,U_1]}(X_{i1}),\dots ,Proj_{[L_D,U_D]}(X_{iD}))$, where:

8 $$\begin{aligned} Proj_{[L_j,U_j]}(X_{ij})= \left\{ \begin{array}{ll} mod(mod(X_{ij},U_j)+U_j,U_j), &{} \text {if } \ j\in \{1,2\},\\ \min (\max (X_{ij},L_j),U_j), &{} \text {if } \ j\in \{3,\dots ,6\},\\ \end{array} \right. \end{aligned}$$

where *mod*(*x*, *y*) is the module function that computes the value of *x* module *y*. Note that we apply the module twice in order to guarantee that the result is a positive value in $[L_j,U_j]=[0, 2\pi ]$, $j\in \{1,2\}$.

Next, for the sake of completeness, we enumerate the steps of the three phases in Fig. 4, namely leader, collaborative and self-guiding.

- **Leader phase**
- Select the leader among the individuals in the current population as follows:
  $$\begin{aligned} {\mathbf {T}}^k=\{{\mathbf {X}}^{k}_i \in P^{k} \mid f({\mathbf {X}}^{k}_i)= \min (f({\mathbf {X}}^{k}_1), \dots , f({\mathbf {X}}^{k}_N))\} \end{aligned}$$
- Compute the mean of each decision variable $j \in \{1,2,\dots ,D\}$ in the population, i.e., $\bar{X^k_j} = \frac{\sum ^N_{i=1} X^k_{ij}}{N}$.
- Generate a leading factor $TF \in \{1,2\}$ randomly.
- Compute a reference point with the following procedure: Given two random numbers $r_1, r_2 \in [0, 1]$, a reference value
  9 $$\begin{aligned} R^k_{ij} = \left\{ \begin{array}{ll} T^k_{j}+r_1^{\frac{1}{n_m+1}-1} \cdot (T^k_j-L_j), &{} \text {if } T^k_j-X^k_{ij}<0 \text { or } \\ &{} (T^k_j-X^k_{ij}=0 \text { and } r_2<0.5),\\ T^k_{j}+(1-r_1^{\frac{1}{n_m+1}}) \cdot (U_j-T^k_j), &{} \text {otherwise,} \end{array} \right. \end{aligned}$$
  is obtained.
- Generate a new candidate value $\tilde{X}^{k}_{ij}$ to replace $X^k_{ij}$:
  $$\begin{aligned} \tilde{X}^{k}_{ij}=X^k_{ij}+{rt}_{ij}^{k} \cdot (T^k_j-TF\cdot \bar{X^k_j}+(TF-1)\cdot R^k_{ij}), \end{aligned}$$
  where ${rt}_{ij}^k\in [0,1]$ is a random number. $\tilde{X}_{ij}^{k}=X_{ij}^{k}$ is maintained for $j=2$ and $j=3$ unless the convergence test is positive.
- $$\tilde{\mathbf {X}}^{k}_{i}=Proj_{\Omega }(\tilde{\mathbf {X}}^{k}_{i})$$.
- Evaluate $$f(\tilde{\mathbf {X}}^{k}_{i})$$.
- If $f(\tilde{\mathbf {X}}^{k}_{i})<f({\mathbf {X}}^k_{i})$, then ${\mathbf {X}}^{k}_{i}=\tilde{\mathbf {X}}^{k}_{i}$ and $f({\mathbf {X}}^{k}_{i})=f(\tilde{\mathbf {X}}^{k}_{i})$.
- **Collaborative phase**
- For each individual in the population $i=1,\dots ,N$, select a different individual by randomly choosing an index $\tilde{i}\in \{1,\dots ,N\}$, $\tilde{i}\ne i$.
- Generate a new candidate value $\tilde{X}^{k}_{ij}$ to replace $X^k_{ij}$:
  $$\begin{aligned} \tilde{X}_{ij}^{k}=X_{ij}^{k}+{rl}_{ij}^k \cdot (X_{\tilde{i}j}^{k}-X_{ij}^{k})\cdot sign(f({\mathbf {X}}_i)-f({\mathbf {X}}_{\tilde{i}})), \end{aligned}$$
  where ${rl}_{ij}^k\in [0,1]$ is a random number and *sign*(*x*) is the sign function:
  $$\begin{aligned} sign(x)= \left\{ \begin{array}{rl} 1, &{} \text {if } x>0, \\ 0, &{} \text {if } x=0, \\ -1, &{} \text {if } x<0. \\ \end{array} \right. \end{aligned}$$
  $\tilde{X}_{ij}^{k}=X_{ij}^{k}$ is maintained for $j=2$ and $j=3$ unless the convergence test is positive.
- $$\tilde{\mathbf {X}}^{k}_{i}=Proj_{\Omega }(\tilde{\mathbf {X}}^{k}_{i})$$.
- Evaluate $$f(\tilde{\mathbf {X}}^{k}_{i})$$.
- If $f(\tilde{\mathbf {X}}^{k}_{i})<f({\mathbf {X}}^k_{i})$, then ${\mathbf {X}}^{k}_{i}=\tilde{\mathbf {X}}^{k}_{i}$ and $f({\mathbf {X}}^{k}_{i})=f(\tilde{\mathbf {X}}^{k}_{i})$.
- **Self-guiding phase**
- Given a random number $r_3\in [0,1]$, a candidate value $\tilde{X}^{k}_{ij}$ to replace $X^k_{ij}$ is obtained as follows:
  10 $$\begin{aligned} \tilde{X}_{ij}^{k}= \left\{ \begin{array}{ll} X_{ij}^{k}+(2r_3)^{\frac{1}{n_m+1}-1}\cdot (X_{ij}^{k}-L_{j}), &{} \text {if } r_3 \le 0.5, \\ X_{ij}^{k}+(1-(2(1-r_3))^{\frac{1}{n_m+1}}\cdot (U_{j}-X_{ij}^{k}), &{} \text {otherwise.} \end{array} \right. \end{aligned}$$
  $\tilde{X}_{ij}^{k}=X_{ij}^{k}$ is maintained for $j=2$ and $j=3$ unless the convergence test is positive.
- $$\tilde{\mathbf {X}}^{k}_{i}=Proj_{\Omega }(\tilde{\mathbf {X}}^{k}_{i})$$
- Evaluate $$f(\tilde{\mathbf {X}}^{k}_{i})$$
- If $f(\tilde{\mathbf {X}}^{k}_{i})<f({\mathbf {X}}^k_{i})$, then ${\mathbf {X}}^{k}_{i}=\tilde{\mathbf {X}}^{k}_{i}$ and $f({\mathbf {X}}^{k}_{i})=f(\tilde{\mathbf {X}}^{k}_{i})$.

During the iterative procedure, some duplicate individuals may appear in the population. Therefore, after the self-learning phase, we implement a mechanism to detect the duplicates and to slightly modify some of their coordinates with a random perturbation (see^27^).

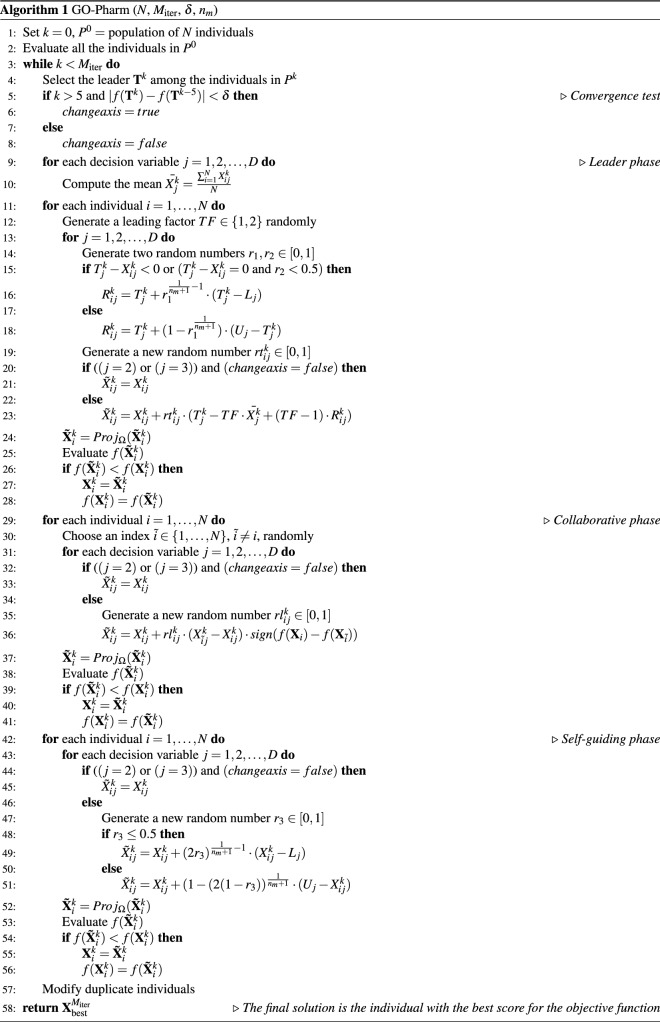



#### Two-layer strategy

To guarantee the exploitation of the search space and avoid premature convergence towards local optima, we design 2L-GO-Pharm using a two-layer strategy^28^.

**Figure 5:**
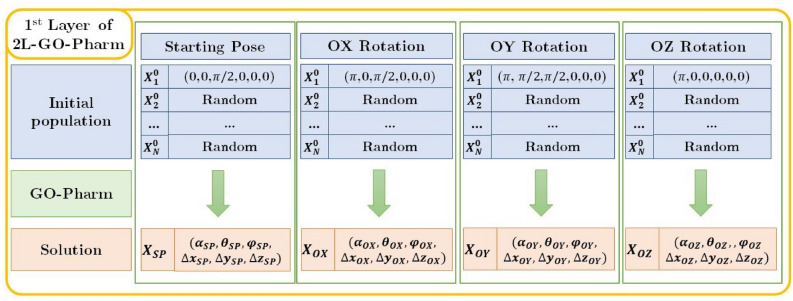
Scheme of the first layer of 2L-GO-Pharm.
- **Second layer of 2L-GO-Pharm** In the second layer, as shown in Fig. 6, the optimization algorithm GO-Pharm is executed one last time considering an initial population that is specifically designed to try to achieve the global optimum. This initial population is composed of:

- **First layer of 2L-GO-Pharm** As represented in Fig. 5, the first layer aims at achieving certain solutions that we will use in the second layer as individuals for the initial population in the optimization algorithm GO-Pharm. To do so, we execute four independent instances of the basic optimization algorithm GO-Pharm with four different initial populations, formed by random individuals and one of the following individuals in each case: the initial or starting pose, the initial pose rotated by $\pi$ radians about the OX-axis, the initial pose rotated by $\pi$ about the OY-axis and the initial pose rotated by $\pi$ about the OZ-axis. Each execution gives us an optimum individual, that we denote by ${\mathbf {X}}_{SP}$, ${\mathbf {X}}_{OX}$, ${\mathbf {X}}_{OY}$, ${\mathbf {X}}_{OZ}$, respectively (see Fig. 5).

**Figure 6:**
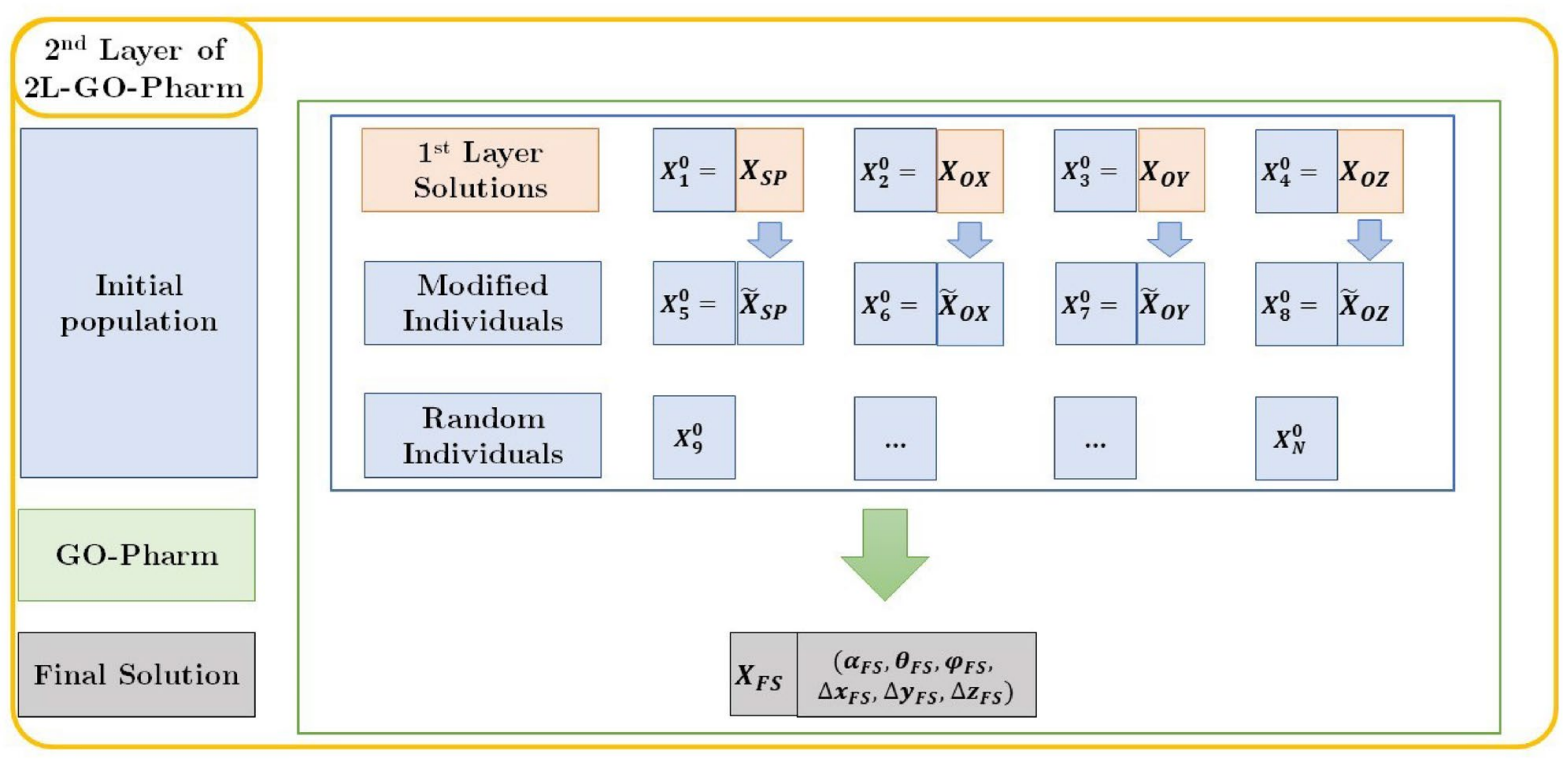
Scheme of the second layer of 2L-GO-Pharm.

- Each one of the four optimum individuals obtained as solutions in the first layer, i.e., ${\mathbf {X}}_{SP}$, ${\mathbf {X}}_{OX}$, ${\mathbf {X}}_{OY}$, ${\mathbf {X}}_{OZ}$.
- Four new individuals $\tilde{\mathbf {X}}_{SP}$, $\tilde{\mathbf {X}}_{OX}$, $\tilde{\mathbf {X}}_{OY}$, $\tilde{\mathbf {X}}_{OZ}$ generated by modifying each one of the previous individuals as follows. Given one of them with its decision variables denoted in general by ${\mathbf {X}}=(\alpha ,\theta ,\varphi , \Delta x, \Delta y, \Delta z)$, we generate a new modified individual $\tilde{{\mathbf {X}}}=(\tilde{\alpha },\tilde{\theta },\tilde{\varphi }, \tilde{\Delta x},\tilde{\Delta y}, \tilde{\Delta z})$, by changing the rotation axis to enhance the exploration of the search space and the diversity of the population:
  $$\begin{aligned} \tilde{\theta }= & {} \theta -\pi /2,\\ \tilde{\varphi }= & {} \pi /2-\varphi . \end{aligned}$$
  Following the same idea of exploration and diversity, the remaining decision variants $\tilde{\alpha }, \tilde{\Delta x},\tilde{\Delta y}, \tilde{\Delta z}$ are randomly generated.
- For the sake of diversity, the remaining individuals to complete the initial population of size *N* are randomly generated.

Finally, considering this new initial population, as we said previously, we execute a last instance of the optimization algorithm GO-Pharm to obtain the final solution (see Fig. 6).

**Figure 7:**
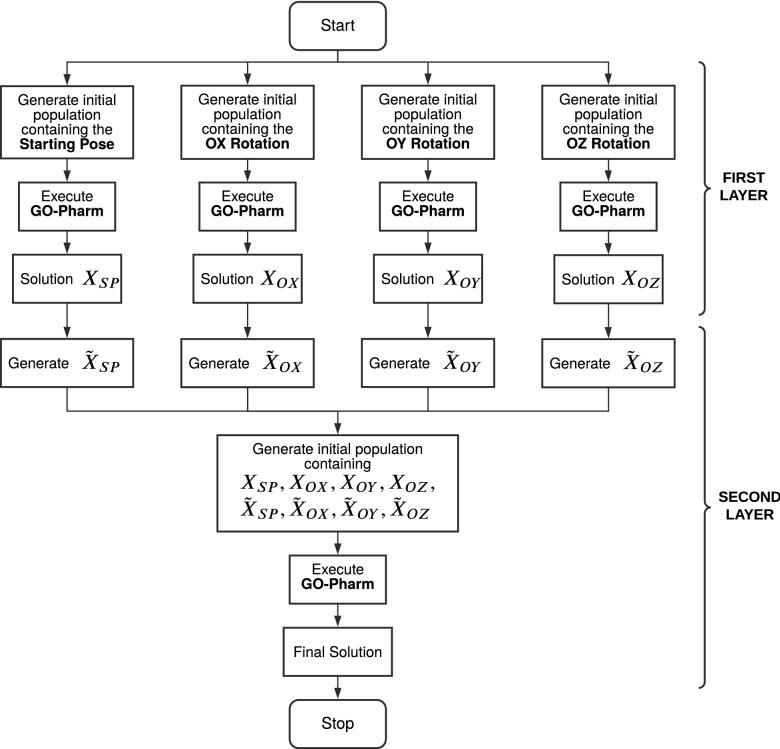
Flux diagram of 2L-GO-Pharm.

In Fig. 7, we show the flux diagram of the 2L-GO-Pharm algorithm, including both layers. Moreover, in Algorithm 2, we detail the pseudocode of 2L-GO-Pharm.

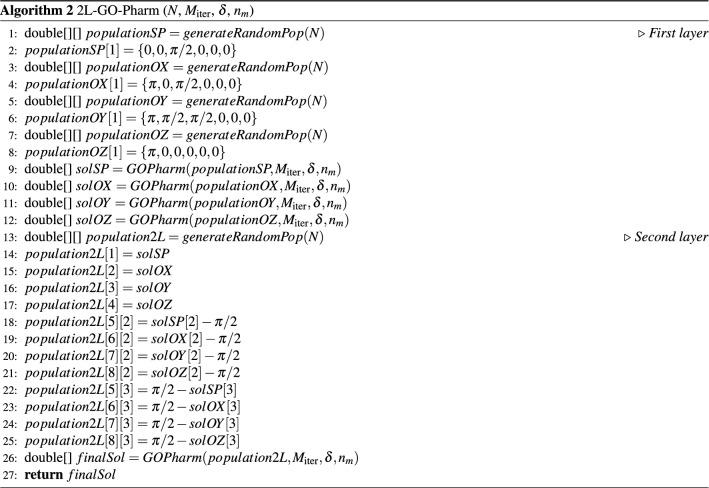



Attending the complexities of their basic operations in one iteration, the complexity of each phase of GO-Pharm (i.e., leader phase, collaborative phase and self-guiding phase) is $O(D\cdot N)$, where *D* is the number of decision variables and *N* is the number of individuals in the population. Therefore, the global complexity of 2L-GO-Pharm is $O(D\cdot N \cdot M_{\mathrm{iter}})$, where $M_\mathrm{iter}$ is the maximum number of iterations.

## Computational experiments framework

To show the performance of 2L-GO-Pharm, we carry out three computational experiments. In the first study, we deal with the shape similarity problem considering the Food and Drug Administration (FDA) database^23^. In the second experiment, we solve the electrostatic similarity problem, also using the FDA data set. Finally, for the third experiment, we compute the AUC classification metric considering the Directory of Useful Decoys (DUD) database^29^, in order to test the ability of the proposed algorithm to distinguish between ligands and decoys by comparing their shape similarity scores.

All the experiments have been executed in a Bullx R424-E3, with 2 Intel Xeon E5 2650v2 (16 cores), 128 GB of RAM memory and 1 TB HDD.

The optimization settings used to configure both OptiPharm and 2L-GO-Pharm algorithms can be consulted in “Optimization settings” section. In “Molecule databases” section, we introduce the two databases involved in the experiments, and, in “The AUC metric” section, we briefly explain the AUC metric.

### Optimization settings

In all computational studies, the OptiPharm configuration considered corresponds to that of OptiPharm Robust (see^14^). More specifically, its input parameters are as follows: 200,000 function evaluations. The number of starting poses is fixed to 5, i.e., (1) the original position of both molecules aligned, (2) rotated the target by $\pi$ radians about the OX-axis, (3) rotated about the OY-axis, (4) rotated about the OZ-axis, (5) randomly generated. The number of iterations is set to 5, i.e., the number of times the reproduction, selection, and optimization mechanisms will be repeated. Finally, the value of the smallest radius of the solutions in the last iterations is set to 1. According to^14^, this configuration is designed to explore better and exploit in-depth the search space to find the optimal possible solution.

In 2L-GO-Pharm, we consider a size population of $N=10$ and a number of iterations of $M_{\mathrm{iter}}=1000$. Then, taking into account that the basic optimization algorithm GO-Pharm evaluates the objective function three times (one evaluation for each of the three phases: leader, collaborative and self-guiding) and that GO-Pharm is executed 5 times (4 in the first layer and 1 in the second layer), it gives a total of $10\times 3\times 1000\times 5=150000$ evaluations. In Eqs. (9) and (10), we set the parameter of the polynomial mutation to $n_m=20$, as suggested in^25^ to obtain a reference point ${\mathbf {R}}^k_{i}$ close to the leader individual ${\mathbf {T}}^k$, and a new candidate point $\tilde{\mathbf {X}}^{k}_{i}$ close to the previous individual ${\mathbf {X}}^{k}_{i}$, respectively. Finally, for the convergence test implemented in 2L-GO-Pharm, we consider a tolerance value of $\delta =$1.0E-4.

OptiPharm and 2L-GO-Pharm are both population-based algorithms but quite different in the processing and managing of the population. In comparison, 2L-GO-Pharm maintains a fixed number of individuals in its population ($N=10$), while in OptiPharm the size of the population changes in each iteration (it starts with 5 individuals and grows in each iteration). Then, to establish a fair comparison, we focus on the number of evaluations of the objective function. Also, we have included in the first layer of 2L-GO-Pharm the problem knowledge used in OptiPharm consisting of the four starting poses, namely the original position of the query and target molecules and the $\pi$ radians rotations about each axis (see Fig. 5).

Both OptiPharm and 2L-GO-Pharm algorithms involve stochasticity since they implement random procedures in their search for new solutions. As a consequence, in order to obtain solid conclusions about their performance, we execute each particular instance 50 times and compute some statistical figures, such as the average and the standard deviation values. Note that this is a common procedure in the literature specialized in heuristic methodologies (see^30^). Once the robustness of a certain algorithm is proved by analyzing the results of those repetitions, it can be used to solve the problem with a single execution providing reliable solutions.

### Molecule databases

In our experiments, we consider two popular databases: the Food and Drug Administration (FDA)^23^ and the Directory of Useful Decoys (DUD)^29^.

The FDA is a federal agency of the United States Department of Health and Human Services. Among other responsibilities, this agency controls the pharmaceutical drugs or medications considered suitable for prescription. Moreover, the FDA supplies a data set that is made up of the medicines approved for safe use in humans in the USA. There are around 2000 FDA-approved drugs that are freely available on the DrugBank database at the time of writing^31^. A frequent benchmark problem is to determine which molecule pairs from the FDA database exhibit high similarity. To deal with that, we use the same subset of 40 query compounds that were considered in^14^ from a total of 1751 FDA molecules. We recall that they were randomly selected as follows. First, the FDA dataset was sorted according to the number of atoms in the compounds. Then, the compounds were separated into 24 intervals and one of their subsets was randomly chosen for each interval, such that the number of selected samples in each interval was proportional to the total of molecules within.

The DUD database contains different sets of compounds. It includes their molecular structure but also some known information about whether they are active or not. This database is widely used to test the efficiency of VS methods for distinguishing ligands that are known to bind to a given protein target, from non-binders or decoys. For each protein of the DUD, active molecules were determined from experimental data. At this point, we should also mention decoys, which correspond to molecules that are physically similar to active compounds, but chemically different, making it difficult for them to act as binders. On average, the DUD includes 36 decoy molecules for each ligand. More details can be found in^14,29^.

Before using any of those databases, we preprocess all their molecules to center and align them following a common guide. First, we place the compounds such that the molecule centroids are at the origin of the coordinates of the search space. Then, we align each molecule by aligning its longest axis with the X-axis and the shortest with the Z-axis.

As was concluded in^14^, considering hydrogen atoms from the compounds in the similarity evaluations, leads to more realistic results. Thus, in this work, we always include the hydrogen atoms in the computations for all the algorithms and all the experiments.

### The AUC metric

The last experiment included in this work is devoted to measuring the ability of the algorithms to distinguish between ligands and decoys. To quantify that, we use the metric known as the Area Under a Curve (AUC).

In general, given a set of compounds, its AUC can be computed by examining a descriptor value that corresponds to each compound. We refer the interested reader to^32^ for a detailed explanation of its calculation. In this case, the considered descriptor is the shape similarity between two molecules given by Eq. (4).

As such, given a query compound, the first step is to solve one by one all the optimization problems consisting of maximizing the shape similarity between this query and each one of the target molecules in the set. As a result, we obtain the shape similarity scores for each query-target pair. Next, we sort the list of target molecules in descending order according to these scores. After that, we compute the AUC value. If it is equal to 1, then the employed methodology allows an accurate distinction between ligands and decoys. It means that it is feasible to find a cut-off point, i.e., a real value that splits the list into two disjoint intervals: one containing all the decoys and another with the ligands. However, this perfect differentiation is not always achieved, in which case, more than two intervals and more cut-off points should be considered. According to this, the AUC value decreases as the number of intervals increases.

## Results and discussion

In this section, we summarize and discuss the main results obtained by 2L-GO-Pharm in comparison with OptiPharm Robust. First, in “Shape similarity results” section, we compare them in terms of shape similarity. Then, in “Electrostatic similarity results” section, we focus on electrostatic similarity. In “Advantages of the two-layer strategy” section, we illustrate the main improvements achieved by using the two-layers scheme. Finally, in “AUC results” section, we include some results comparing the area under the curve.

### Shape similarity results

In the first computational experiment, we consider 40 query molecules from the FDA database, which were selected as explained in “Molecule databases” section. Then, for each query, we study which molecules (i.e., targets) from the FDA database are the most similar to that query, regarding their shape similarity. To do that, we solve the problem of maximizing the shape similarity of each query-target pair. Note that in those studies, we discard the trivial case where query and target are the same molecules.

**Figure 8:**
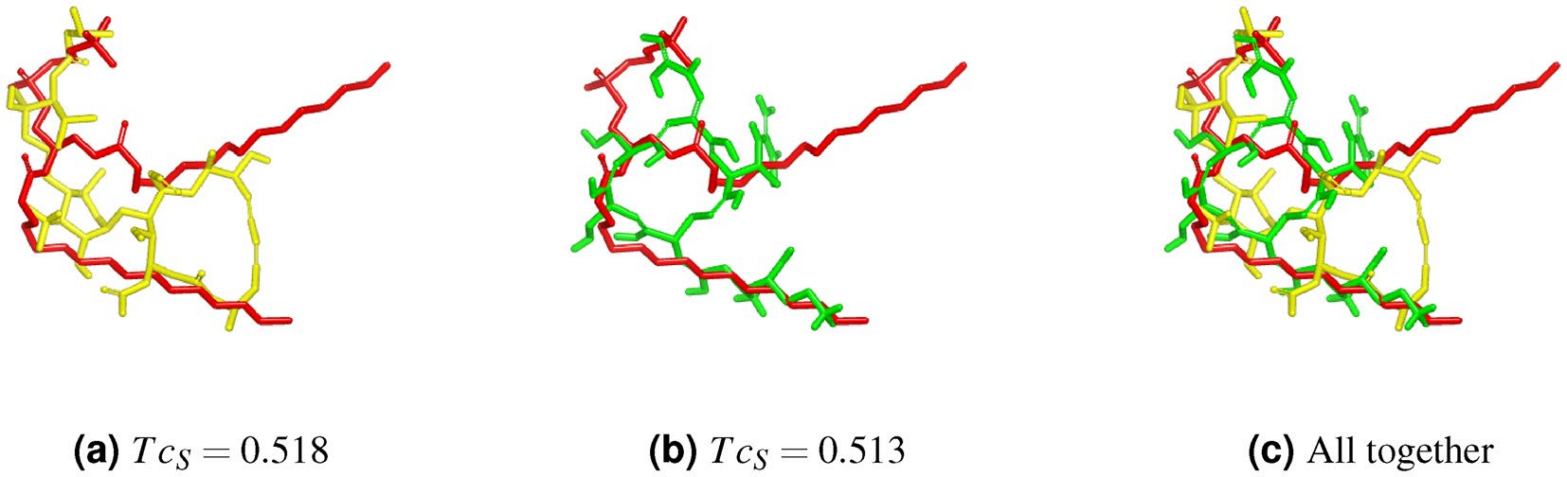
Query compound DB09114 is represented by the red structure. Colours remain fixed. (**a**) $Tc_{S}=0.518$ where the compound DB01321 is the yellow structure. (**b**) $Tc_{S}=0.513$ where the compound DB08993 is the green structure. (**c**) The two previous compounds are optimized with respect to the query.

Concerning shape similarity, the optimum values $Tc_{S}$ obtained by 2L-GO-Pharm and OptiPharm Robust are quite similar. In fact, when we list the compounds in descending order of shape similarity, the most similar compound, which is listed in first place, is the same for both algorithms in 39 of the 40 cases. There is one case in which 2L-GO-Pharm found a different target to be the most similar: the query compound DB09114. For this query, OptiPharm Robust found DB08993 to be the most similar compound in the database, with $Tc_{S}=0.512$ (see^14^). However, 2L-GO-Pharm is able to find the target compound DB01321 with $Tc_{S}=0.518$, which has a higher value of shape similarity. The compound DB08993 found by OptiPharm is placed second in the 2L-GO-Pharm list of most similar compounds, with $Tc_{S}=0.513$. In Fig. 8, we represent the query molecule DB09114 in red and the first and second most similar compounds found by 2L-GO-Pharm, DB01321 in yellow and DB08993 green, respectively.

Thus, according to our results, 2L-GO-Pharm, with a sizeable number of evaluations lower than OptiPharm, achieves shape similarity scores that are very similar or even better than those obtained by OptiPharm. More precisely, in these experiments, 2L-GO-Pharm is configured to use 50000 fewer evaluations of the objective function than OptiPharm in each execution query-target. Thus, taking into account that the considered database has 1750 molecules in total (without including the query compound), the savings in evaluations is 87.5 million for each query.

### Electrostatic similarity results

Here we show and discuss the results of the second computational experiment, where the same 40 query molecules from the FDA database are considered. Analogously to the previous experiment, for each query, we study which target molecules from the FDA database are the most similar to that query, but now focusing on their electrostatic similarity. We then solve the problem of maximizing the electrostatic similarity for all the query-target combinations, where the target molecule is not the same as the query.

In Table 1, we include, for each query, the target with the best value for electrostatic similarity ($Tc_{E}$) found by OptiPharm Robust (see^15^) and the one obtained by 2L-GO-Pharm. Moreover, in the last column, we indicate the ranking or position of the OptiPharm target in the list of targets found by 2L-GO-Pharm ordered from the most to the least similar. We can observe that:

- the most similar compound obtained by OptiPharm is not always the most similar found by 2L-GO-Pharm;
- the values of electrostatic similarity in 2L-GO-Pharm are, in general, higher than the ones in OptiPharm;
- on average, OptiPharm obtains an electrostatic similarity score of 0.691 and 2L-GO-Pharm obtains 0.708, which indicates slightly higher values of similarity;
- on average, the compounds found by OptiPharm are located between second and third position in the 2L-GO-Pharm ranking of the most similar targets.

**Table 1:** Results obtained for 40 query compounds from the FDA database.

Query	OptiPharm		2L-GO-Pharm		Ranking
	Target	$Tc_{E}$	Target	$Tc_{E}$	Rk
DB00529	DB00818	0.720	DB00818	0.731	1
DB00331	DB01018	0.790	DB01018	0.810	1
DB01365	DB01626	0.964	DB06706	0.969	4
DB01352	DB00306	0.983	DB00306	0.986	1
DB00380	DB08971	0.505	DB08971	0.532	1
DB00674	DB00514	0.662	DB00405	0.699	4
DB00632	DB00898	0.997	DB00898	0.997	1
DB07615	DB09218	0.892	DB09218	0.896	1
DB00693	DB00692	0.454	DB00765	0.484	5
DB00887	DB01127	0.662	DB01127	0.659	1
DB09219	DB00316	0.450	DB09236	0.502	4
DB00381	DB00630	0.377	DB01023	0.432	6
DB09237	DB08998	0.902	DB08998	0.919	1
DB01198	DB00123	0.894	DB00123	0.897	1
DB00876	DB00774	0.532	DB00774	0.572	1
DB01621	DB04861	0.867	DB08897	0.868	10
DB09236	DB00449	0.664	DB00449	0.681	1
DB08903	DB01359	0.888	DB01227	0.897	2
DB00728	DB09131	0.874	DB00728	0.883	1
DB01419	DB01611	0.933	DB01611	0.938	1
DB00320	DB00120	0.563	DB00120	0.578	1
DB01232	DB09089	0.791	DB09089	0.793	1
DB00246	DB05271	0.877	DB09019	0.896	7
DB00503	DB01144	0.401	DB00615	0.468	3
DB09114	DB00583	0.876	DB00583	0.878	1
DB00254	DB00271	0.836	DB00271	0.847	1
DB00309	DB00319	0.467	DB00319	0.476	1
DB06439	DB00878	0.488	DB08965	0.529	4
DB01196	DB08797	0.527	DB00828	0.547	7
DB01078	DB01085	0.540	DB01085	0.565	1
DB01590	DB00653	0.529	DB00653	0.536	1
DB04894	DB09131	0.662	DB09131	0.669	1
DB04786	DB09159	0.910	DB09138	0.919	3
DB00732	DB00653	0.508	DB01333	0.535	2
DB00403	DB06335	0.575	DB06335	0.602	1
DB00050	DB00516	0.385	DB06274	0.409	7
DB06699	DB09131	0.642	DB09131	0.656	1
DB06219	DB09131	0.670	DB09131	0.654	1
Mean	–	0.691	–	0.708	2.4

For each query, the target with the best value of electrostatic similarity ($Tc_{E}$) found by OptiPharm Robust (see^15^) and the one obtained by 2L-GO-Pharm are reported. In the last column (Ranking), we indicate the position of the OptiPharm target in the list of targets found by 2L-GO-Pharm ordered from the most to the least similar.

**Figure 9:**
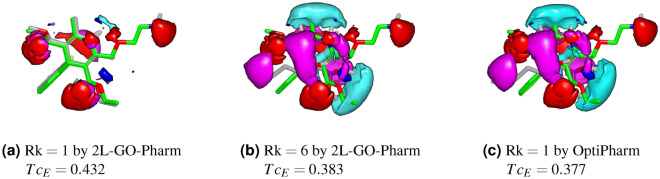
Query compound DB00381 is coloured green. Query electrostatic fields are coloured deep blue and red. Target compounds are shown in grey and their electrostatic potential fields, in light blue and pink. (**a**) $Tc_{E}=0.432$ where the compound DB01023 is the most similar ($\mathrm{Rk}=1$) in electrostatics found by 2L-GO-Pharm . (**b**) $Tc_{E}=0.383$ where the compound DB00630 is sixth ($\mathrm{Rk}=6$) in the list of most similar compounds according to 2L-GO-Pharm. (**c**) $Tc_{E}=0.377$ where the compound DB00630 is first ($\mathrm{Rk}=1$) in the list of most similar compounds according to OptiPharm.

As an example case, we focus on the query molecule DB00381. As can be seen in Table 1, row 12, OptiPharm finds the compound DB00630 to be the most similar in terms of electrostatic potential with a score of $Tc_{E}=0.377$. However, 2L-GO-Pharm enhances this similarity value obtaining $Tc_{E}=0.432$ for DB01023 but also finds five different compounds with a higher score. In Fig. 9, we show the query compound DB00381 in green with its electrostatic potential field in dark blue and red. In Fig. 9a, we compare this query DB00381 with the target molecule DB01023, which obtains the best value of electrostatic similarity according to 2L-GO-Pharm, $Tc_{E}=0.432$. Target compounds are shown in grey and their electrostatic potential fields, in light blue and pink. After that, in Fig. 9b, we compare it with the target molecule DB00630, which is placed sixth in the list of most similar compounds in electrostatics according to 2L-GO-Pharm, with $Tc_{E}=0.383$. Finally, in Fig. 9c, we also compare the query with the target molecule DB00630, but in the position of best electrostatic similarity found by OptiPharm that gives $Tc_{E}=0.377$. This example shown in Fig. 9 illustrates that 2L-GO-Pharm is able to obtain compounds and electrostatic similarity scores which improve the results found by OptiPharm Robust. Moreover, 2L-GO-Pharm achieves it by performing a lower number of evaluations. As stated in the previous section, for the current database, 2L-GO-Pharm performs 87.5 million evaluations less than OptiPharm when processing a query.

While, in shape similarity, their results were practically the same regarding the similarity scores (see “Shape similarity results” section), for the electrostatic potential similarity, we notice that 2L-GO-Pharm significantly outperforms OptiPharm. It highlights the ability of 2L-GO-Pharm to explore the search space avoiding being trapped in local solutions and its relevance when dealing with non-smooth objective functions, as is the case with electrostatic similarity (see “Smoothness of the objective functions” section).

### Advantages of the two-layer strategy

This section is devoted to highlighting how the two-layer strategy prevents getting trapped in some local optimum, enhances the exploration of the search space, and, finally, improves the accuracy of the results. To show our point, we cite some particular examples.

For instance, regarding shape similarity between the query DB09236 and the target DB01054, in a single execution of our 2L-GO-Pharm algorithm, it found, in the first layer, a score of 0.571 from the population including the initial pose, 0.681 from the population including the initial pose rotated by $\pi$ radians about the OX-axis, 0.571 from the one including the initial pose rotated by $\pi$ about the OY-axis and 0.681 from the population with the initial pose rotated by $\pi$ about the OZ-axis. Next, the second layer of 2L-GO-Pharm collected all these solutions and gave us a final score of 0.682. This particular instance shows us that in two cases, the GO-Pharm algorithm, using it by itself without the two-layer strategy, gets trapped into a local minimum which gives the score of 0.571, and in the other two cases, it needs a little more exploitation to improve the final solution.

Studying the algorithm’s performance for different runs to deal with stochasticity, we found that getting trapped into a local minimum does not always occur starting from a particular population with a concrete pose. Sometimes it happens with the population including the initial pose, but sometimes it occurs with the population including the initial pose rotated by $\pi$ radians about the OX-axis or any other populations. Even we observe that in some runs, all the minima found in the first layer are local, and this was the reason to include in the second layer’s population four more individuals derived from the first layer solutions as follows. For each optimum individual ${\mathbf {X}}=(\alpha ,\theta ,\varphi , \Delta x, \Delta y, \Delta z)$ resulting from the first layer, we obtain a new individual: $\tilde{{\mathbf {X}}}=(\tilde{\alpha },\tilde{\theta },\tilde{\varphi }, \tilde{\Delta x},\tilde{\Delta y}, \tilde{\Delta z})$, where $\tilde{\theta }=\theta -\pi /2$, $\tilde{\varphi }=\pi /2-\varphi$, and the remaining decision variants $\tilde{\alpha }, \tilde{\Delta x},\tilde{\Delta y}, \tilde{\Delta z}$ are randomly generated (see “Two-layer strategy” section).

Concerning electrostatic similarity, we can also give an illustrative example. For instance, considering the query DB00381 and the target DB01023, in a single execution of our 2L-GO-Pharm algorithm, it found, in the first layer, scores of 0.256, 0.422, 0.430, and 0.258 starting from the population including the initial pose, from the population including the initial pose rotated by $\pi$ radians about the OX-axis, from the one including the initial pose rotated by $\pi$ about the OY-axis and from the population with the initial pose rotated by $\pi$ about the OZ-axis, respectively. Finally, in the second layer, it achieves a final score of 0.432, which is a more accurate solution.

### AUC results

In Table 2, for each query on the DUD database, the average (Av) and the standard deviation (SD) of the AUC values obtained over 50 independent runs by OptiPharm Robust and by 2L-GO-Pharm are included. In addition, in the last row, we compute the average values considering all the query molecules. We can then observe that:

- the average 2L-GO-Pharm AUC values are, in general, slightly higher than the OptiPharm ones;
- on average, OptiPharm obtains an AUC value of 0.65 and 2L-GO-Pharm obtains 0.66, which indicates very similar or even slightly higher values for AUC;
- on average, OptiPharm obtains an SD value of 0.005 and 2L-GO-Pharm achieves 0.003, which seems to indicate that 2L-GO-Pharm is a little more robust than OptiPharm.

**Table 2:** AUC for DUD database with hydrogens.

Query	OptiPharm		2L-GO-Pharm	
	Av	SD	Av	SD
ace	0.40	0.001	0.40	0.004
ache	0.72	0.002	0.74	0.002
ada	0.79	0.006	0.79	0.001
alr2	0.46	0.007	0.50	0.004
ampc	0.74	0.013	0.76	0.009
ar	0.86	0.003	0.86	0.001
cdk2	0.62	0.003	0.62	0.003
comt	0.40	0.008	0.38	0.004
cox1	0.59	0.001	0.58	0.001
cox2	0.90	0.001	0.91	0.001
dhfr	0.59	0.004	0.59	0.002
egfr	0.56	0.002	0.56	0.001
er_agonist	0.74	0.003	0.74	0.001
er_antagonist	0.69	0.004	0.69	0.005
fgfr1	0.42	0.000	0.42	0.001
fxa	0.66	0.009	0.74	0.003
gart	0.28	0.011	0.29	0.004
gpb	0.85	0.002	0.86	0.001
gr	0.77	0.004	0.77	0.004
hivpr	0.74	0.01	0.77	0.004
hivrt	0.70	0.008	0.73	0.003
hmga	0.84	0.004	0.87	0.002
hsp90	0.77	0.012	0.83	0.003
inha	0.59	0.010	0.61	0.005
mr	0.87	0.003	0.87	0.001
na	0.83	0.002	0.84	0.002
p38	0.31	0.004	0.30	0.002
parp	0.59	0.004	0.59	0.002
pde5	0.77	0.006	0.79	0.002
pdgfrb	0.44	0.004	0.44	0.002
pnp	0.71	0.004	0.73	0.002
ppar_gamma	0.73	0.006	0.73	0.003
pr	0.68	0.011	0.69	0.004
rxr_alpha	0.89	0.023	0.96	0.003
sahh	0.88	0.006	0.9	0.002
src	0.44	0.002	0.44	0.001
thrombin	0.56	0.010	0.57	0.004
tk	0.65	0.003	0.66	0.002
trypsin	0.27	0.004	0.26	0.002
vegfr2	0.62	0.003	0.61	0.003
Mean	0.65	0.005	0.66	0.003

For each query compound, the average (Av) AUC value and the standard deviation (SD) over 50 independent executions were computed with both OptiPharm Robust and 2L-GO-Pharm. The last row of the table shows average values for all the considered query molecules.

As in the previous experiments, 2L-GO-Pharm is configured to use 50000 fewer evaluations of the objective function than OptiPharm in each execution query-target. Therefore, since the DUD database has 28374 molecules in total, the savings in evaluations is 6418.7 million for each query. In resume, 2L-GO-Pharm performs equal or even better than OptiPharm in terms of AUC but with less computational effort.

## Conclusions

In this work, we propose a new algorithm for VS called 2L-GO-Pharm, consisting of two layers and a core mono-objective evolutionary algorithm named GO-Pharm. Though the well-known TLBO algorithm inspires it, it incorporates mechanisms of interest that are original and that enhance the search for compounds similar to a given one. The specific conclusions are as follows:

- For the shape problem, 2L-GO-Pharm results are similar to those of OptiPharm. Indeed, in 39 of the 40 queries tested, the most similar compound found by both algorithms matches. However, there is one case where 2L-GO-Pharm found another target to be the most similar with a slightly higher value of shape similarity.
- For the electrostatic similarity problem, 2L-GO-Pharm generally achieves better similarity results than OptiPharm, showing a higher average score. Furthermore, it can be observed that the 2L-GO-Pharm improvements are more noticeable and significant than shape similarity. It can be explained by the fact that the electrostatic similarity is a non-smooth function with a great number of peaks and that 2L-GO-Pharm is specifically designed to avoid getting trapped in local optima.
- Regarding the AUC results, we can state that 2L-GO-Pharm is a robust algorithm that also slightly improves OptiPharm for AUC averages.
- Finally, a significant reduction in computational time can be observed in all experiments. More precisely, in the experiments performed on the FDA database, 2L-GO-Pharm saves 87.5 million evaluations per query. For the DUD evaluation, 2L-GO-Pharm needs 6418.7 million fewer evaluations.

Considering all these results, we can conclude that 2L-GO-Pharm is a better alternative to OptiPharm in terms of solution quality and computational cost savings. The latter is especially important due to the large databases that must be processed.

In future work, we will deal with VS problems where the molecules are flexible. For those problems, 2L-GO-Pharm could make a significant difference since the databases increase their size considerably and the number of variables could increase, thereby increasing the dimensions of the search space.
